# Effects of Oat Hay Content in Diets on Nutrient Metabolism and the Rumen Microflora in Sheep

**DOI:** 10.3390/ani10122341

**Published:** 2020-12-09

**Authors:** Xuejiao An, Lingyun Zhang, Jing Luo, Shengguo Zhao, Ting Jiao

**Affiliations:** 1 College of Animal Science and Technology, Gansu Agricultural University, Lanzhou 730070, China; 18393811056@163.com (X.A.); zhanglingyun1110@163.com (L.Z.); luojing1286367055@163.com (J.L.); 2 Key Laboratory of Grassland Ecosystem, Gansu Agricultural University, Lanzhou 730070, China; 3 College of Grassland Science, Gansu Agricultural University, Lanzhou 730070, China

**Keywords:** oat hay, 16S rRNA, microorganisms, sheep

## Abstract

**Simple Summary:**

Providing high-quality forage for the animal husbandry to improve the quality of animal husbandry products and meet the needs of the public is one of the focuses of the current animal husbandry development. Oats have high nutritional value, and the planting area in our country is increasing year by year. It has become the new favorite of the breeding industry, and its development prospects are very broad. At present, there are few domestic research reports on the best addition of oat hay in ruminant diets, ruminant digestion and metabolism, and ruminant flora. Therefore, the purpose of this study is to add different proportions of oat hay to sheep feed to study its effect on sheep digestion and metabolism and rumen microflora. The experimentally obtained data showed that, with the increase in the content of oat hay in the diet, the appearance of sheep dry matter (DM), organic matter (OM), acid detergent fiber (ADF), and neutral detergent fiber (NDF) digestibility, as well as the diversity and abundance of rumen microbes, showed an upward trend. All presented data provide a theoretical basis for the scientific application of oat hay in sheep breeding.

**Abstract:**

Oats have the characteristics of drought tolerance, cold resistance, strong adaptability, high forage yield, and high nutritional value. However, there are few reports on the most appropriate amount of oat hay in ruminant diets, the digestion and metabolism of ruminants, and the rumen microflora. To study the effects of oat hay content in diets on nutrient digestion and metabolism and the rumen microflora in sheep, 9 German Merino and Mongolian crossbred rams of similar body condition and weight with permanent fistulas were selected. The 3 × 3 Latin square design was used to randomly divide the rams into 3 groups, with 3 animals in each group. The three groups were fed different kinds of roughage: whole-plant corn silage only (corn silage group, CSG), oat hay mixed with whole-plant corn silage (1:1) (mixed group, MG), and oat hay only (oat hay group, OHG). The nutrient digestion and metabolism of each group were measured, and the pH and rumen microflora were examined after feeding for different durations. Dynamic changes in microbial communities were detected. The nutrient digestion and metabolism results showed that, with an increase in the content of oat hay in the diet, the intake and apparent digestibility of dry matter (DM) and organic matter (OM) showed an increasing trend, and the intake, digestion, and stability of acid detergent fiber (ADF) and neutral detergent fiber (NDF) increased in the OHG. The apparent digestibility, dietary nitrogen, deposited nitrogen, and nitrogen retention rate in this group were significantly higher than those in the CSG (*p* < 0.05). The rumen pH and sequencing results showed that the rumen fluid pH of the CSG was significantly lower than that of the OHG at 1 and 5 h (*p* < 0.05). The main microbial in the rumen of the three groups of sheep were Bacteroides, Sclerotium, and Proteus. The dominant taxon in the CSG was Prevotella, followed by *Vibrio syringae*, and the dominant taxon in the MG and OHG was Prevotella, followed by Rikenellaceae. Redundancy analysis showed that ADF and NDF in the feed had an effect on the abundance of Fibrobacteres, Ruminococcaceae, and Prevotella. Our findings indicate that the use of oat hay roughage in the diet significantly improves the apparent digestibility of NDF and ADF and helps maintain the stable state of the sheep’s rumen internal environment and the growth of rumen microorganisms.

## 1. Introduction

Oat (*Avena sativa* L.) is the main annual forage cultivated on the Qinghai-Tibetan Plateau, where it is well adapted to the harsh alpine environment, and is one of the most widely grown annual cool-season forage cereals in the world [1,2]. As an annual forage, oat has a relatively low cost, it is easy to manage, and can be included in various crop rotations [3]. Oat has become an important forage for livestock, especially as a supplementary feed during winter [4,5]. George et al. reported that used oat hay as the only dietary component for beef cattle revealed that the excreted nitrogen and deposited nitrogen decreased significantly as the feeding level decreased [6]. Long et al. fed yaks oat hay with free intakes of 30%, 60%, and 90%, and the dry matter (DM) digestibility decreased with an increasing oat hay content [7]. A study on the use of oat hay to feed hybrid beef cattle showed that, when the oat hay level dropped from 95% to 40%, feed digestibility was significantly reduced [8]. A study on the methane (CH4) production and nutrient utilization potential of oat grass and silage in *Mulla buffalo* revealed that the intake of fiber (neutral detergent fiber, NDF; acid detergent fiber, ADF) in the hay-fed group was significantly higher than that in the control group [9]. Feeding weaned calves with oat hay can improve rumen fermentation and nitrogen utilization, as well as reduce incidence of diarrhea in post-weaning dairy calves [10].

There are many kinds of microorganisms, mainly including bacteria, anaerobic fungi, protozoa, and archaea, in the rumen of ruminants [11]. These microorganisms can convert feed fiber in the rumen into compounds that are easily digested and absorbed by the body and can then be utilized [12]. During the long-term selection and evolutionary process, these microorganisms formed a mutually dependent relationship with the host. This relationship plays a vital role in maintaining the health of ruminants and improving production performance [13,14]. Studies have shown that the composition and abundance of rumen microbes are affected by many factors, such as individual differences among animals [15], the composition of diets, and the ratio of fineness to roughness [16]. A study on the effects of dried oats on the growth performance of dairy cows before and after weaning, measured via rumen fermentation and biochemical blood indicators, revealed that starter feed containing chopped oat hay could improve rumen fermentation parameters, which might allow successful transition from a pre-ruminant to mature ruminant state [17]. The oat hay supplementation could improve the pH of the calf’s rumen by changing the ratio of various microbial populations [18]. Although oat hay has been widely used as roughage in sheep and dairy cattle breeding in China, there have been few domestic research reports on the optimal addition of oat grass, its digestion and metabolism, rumen fermentation, and related topics, which complicates production practices. Therefore, in this study, by feeding sheep different contents of oat hay to study its effects on the digestion and metabolism of nutrients and the rumen microflora, the optimal supplementation of oat hay in the sheep’s diet was selected to improve the sheep’s diet. The utilization rate of coarse feed provided a theoretical basis for the scientific breeding of sheep.

## 2. Materials and Methods

### 2.1. Experimental Design

All experiments involving animals were reviewed and approved by the Animal Committee of Gansu Agricultural University (GSAU-2015-0089) on 10 Oct, 2015. A total of 9 German Merino and Mongolian sheep hybrid rams with similar body weights {(70.32 ± 2.14) kg} were selected. Nine sheep were randomly divided into 3 groups fed with whole corn silage (CSG), oat hay mixed with whole-plant corn silage (MG), and dried oat hay (OHG); using a 3 replicated 3 × 3 Latin square, designs were used to carry out phase 3 digestion and metabolism tests (Table 1). Each period was 15 days, where the first 10 days were the pre-feeding period and the last 5 days were the sampling period.

### 2.2. Experimental Animal Feeding and Management

Three months prior to the start of the experiment, 9 healthy German Merino and Mongolian sheep brucellosis negative rams were surgically fitted with a permanent rumen fistula (Gansu Agricultural University, Lanzhou, China). After surgery, the rams were fed a supplement formulated for the prevention of parasites and disease, while regaining and maintaining body condition, during post-surgical healing period. Each test sheep’s body surface, walkway, trough, sink, and sheep house were sprayed with peroxyacetic acid every week, and the ground was disinfected with lime. In the pre-feeding period, the test sheep were kept in a single pen and were uniformly dewormed before entering the house. The sheep house was sprayed and disinfected with a 3% NaOH solution. At the initiation of the experiment, the fistulated rams were blocked by body weight and age and randomly divided into 3 groups, the five-day sampling period was raised in metabolic cages, and the 10-day adaptation period is in feedlot.

The roughage was whole corn silage and oat hay. Oat hay was purchased from Xiahe County of Gannan Prefecture in China and made 3–5 cm in length by a straw cutter. The concentrate was mainly corn, soybean meal, rapeseed meal, and 1% premix. The composition and nutritional levels of the diet are shown in Table 2. The sheep were fed the same amount twice a day at 08:00 and 18:00 and allowed to drink freely. During the test, the daily feed intake of each sheep was recorded, and the remaining material was weighed and recorded before each feeding.

### 2.3. Collection and Processing of Experimental Samples

#### 2.3.1. Collection and Processing of Feed Samples

In each period of the experiment, before feeding at 8:00 and 18:00 every day, the remaining feed was collected, and a small amount of mixed concentrate and coarse material samples were collected. After mixing, samples were taken by the quadruple method and pulverized through a 0.45-mm sieve to analyze nutrients, such as dry matter (DM), crude protein (CP), neutral detergent fiber (NDF), and acid detergent fiber (ADF) in the diet samples.

#### 2.3.2. Collection and Treatment of Fecal and Urine Samples

The fecal samples were collected at 8:00 on the first day of the trial period; then, they were collected once in the morning and evening and continuously for 5 days. Immediately after sputum, the fresh weight was determined, 10% of the total weight was taken as a sample, and the sample was naturally dried at room temperature. After weighing and recording, we mixed the three-day sample in equal amounts; one part was used to measure fecal nitrogen, and the other part was used to determine the initial moisture. The sample was smashed through a 40-mesh sieve and stored at 4 °C to prepare nutrients, such as DM, CP, NDF, and ADF. After collecting the urine sample, 5 mol/L 10% hydrochloric acid was added at 1:50 (acid:urine), and a sample was taken every 6 h. The sample was mixed for five days, and 5% was taken and stored at −20 °C. Urine nitrogen (UN) was measured.

#### 2.3.3. Rumen pH Measurement and Microbial Sample Collection

Nine experimental sheep were randomly divided into 3 groups, and 3 repeated 3 × 3 Latin square designs were used to carry out 3 feeding trials for 15 days each, with the first 10 days being the pre-feeding period and the next 5 days being the sampling period. Rumen fluid was collected at 0 (before feeding), 1, 3, 5, and 7 h on the last day of each trial, and the pH of the rumen fluid was measured, after which the fluid was stored at −80 °C.

### 2.4. Experimental Methods

#### 2.4.1. Determination of Feed Nutrient Content

The contents of DM, CP, Ca, P, NDF, ADF, and UN in feed and manure were measured by conventional methods [19]. We used the following formula to calculate the experimental results:

Feed intake (kg/d) = feed amount (kg/d) − residual feed (kg/d);

Nutrient digestion (g/d) = nutrient intake (g/d) − fecal volume (g/d);

Apparent digestibility of nutrients (%) = (nutrient intake (g/d) − the nutrient quality in the feces (g/d)/nutrient intake (g/d) × 100%;

Sedimentary nitrogen (g/d) = ingested nitrogen (g/d) − fecal nitrogen (g/d) − urine nitrogen (g/d);

Nitrogen retention rate (%) = sedimentary nitrogen (g/d)/intake nitrogen (g/d) × 100%.

#### 2.4.2. DNA Extraction and HiSeq Sequencing

Total rumen microbes DNA was extracted from each sample using NucleoSpin^®^ Soil extraction kit (MACHEREY-NAGEL, 740780.50, Dueren, North Rhine Westphalia, Germany) according to the manufacturer’s instructions, and the concentrations and quality of DNA samples were examined by using a NanoDrop 2000 (Thermo Scientific, Waltham, MA, USA). Then, the samples were sent to Yuan Quan Yi Ke Biotechnology Co., Ltd. (Beijing, China), where the Illumina HiSeq platform (San Diego, CA, USA) was used for sequencing.

### 2.5. Bioinformatic Analysis

The raw pyrosequencing reads were sorted with barcodes and quality trimmed using QIIME 1.8.0 [20]. The parameters were as follows: paired-end reads with at least a 50-bp overlap and <5% mismatches were combined using FLASH [21]. An average quality score threshold of >30 over a 5-bp window size was used to trim the unqualified sequences using BTRIM [22]. Any joined sequences with ambiguous bases and lengths < 200 bp were discarded. All rarefaction curves approached a plateau, suggesting that the sequencing depth of all samples was sufficient to cover microorganismal community diversity. Sequences were clustered into operational taxonomic units (OTUs) with a 97% identity threshold using UPARSE [23], with the chimeras and all singletons being discarded. We determined taxonomic annotations using the Ribosomal Database Project Classifier with an 80% confidence score [24]. The 16S sequences were assigned using the Silva database [25].

### 2.6. Statistical Analyses

Alpha diversity metrics, including the Shannon-Wiener index and Simpson diversity index, were calculated using the “diversity” function in the vegan package (version 2.4.4) of R software version 3.3.2 (Lucent Technologies, New Jersey, NJ, USA). microbial community structure was visualized by nonmetric multidimensional scaling (NMDS) ordination based on Bray-Curtis dissimilarity matrices using the vegan package in R software. One-way analysis of variance (ANOVA) was used to examine changes in the copy number of microbial genomes in the same group at different time periods or among different groups. Pearson correlation coefficients were used to assess the associations between microbial alpha diversity and environmental factors and the relationships between various environmental factors. The linear discriminant analysis (LDA) effect size (LEfSe) technique was performed to reveal the significant ranking of abundant modules in different samples. All statistical analyses were conducted with SPSS 22.0 software (SPSS Inc., Chicago, IL, USA). The data analysis method adopted in ANOVAs was the least significant difference (LSD) method. The results are expressed as the mean ± standard error. Values of *p* < 0.05 were considered statistically significant, and values of *p* < 0.01 were considered extremely statistically significant.

## 3. Results

### 3.1. Effects of Oat Hay Content in the Diet on the Apparent Digestibility of DM and OM in Sheep

Table 3 shows that the content of oat hay in the diet had no significant effect on the intake, fecal output, digestion, and apparent digestibility of dry matter (DM) (*p* > 0.05). However, in addition to the amount of DM excreted, the DM intake and digestion of the groups fed a mixture of oat hay and corn silage (MG) or oat hay only (OHG) were higher than those of the group fed corn silage only (CSG). The organic matter (OM) intake of the CSG was lower than that of the MG and significantly lower than that of the OHG (*p* < 0.05). The excretion of OM in the feces was significantly higher in the CSG than in the OHG (*p* < 0.05).

### 3.2. Effects of Oat Hay Content in the Diet on the Apparent Digestibility of NDF and ADF in Sheep

Table 4 shows that the intake, digestion, and apparent digestion of NDF in sheep increased with an increasing oat content, and the intake of NDF in the OHG was extremely significantly higher than that in the CSG (*p* < 0.01). The digestibility and apparent digestibility in the OHG were significantly higher than those in the CSG (*p* < 0.05). Fecal output decreased with an increasing oat hay content, and the fecal output of NDF in the OHG was significantly lower than that in the CSG (*p* < 0.05). The trends of the intake, fecal excretion, digestion, and apparent digestibility of sheep ADF were basically consistent with those of NDF. The fecal excretion of ADF in the OHG was significantly lower than that in the CSG (*p* < 0.05), and the digestibility and apparent digestibility in the OHG were significantly higher than those in the CSG (*p* < 0.05).

### 3.3. Effect of Oat Hay Content in Feed on the Apparent Digestibility of Nitrogen in Sheep

Table 5 shows that the content of oat hay in the diet had no significant effect on the digestibility and apparent digestibility of fecal nitrogen, UN, and nitrogen in sheep. However, with an increase in oat hay content, the intake, digestibility and apparent digestibility of nitrogen increased, with the groups ranking as follows: OHG > MG > CSG. Moreover, the intake of nitrogen in the OHG was significantly higher than that in the CSG (*p* < 0.05). The sedimentation rate of nitrogen and nitrogen deposition increased significantly with the oat hay content (*p* < 0.05).

### 3.4. The Effect of Oat Hay Content in the Diet on the Rumen pH of Sheep

**Figure 1:**
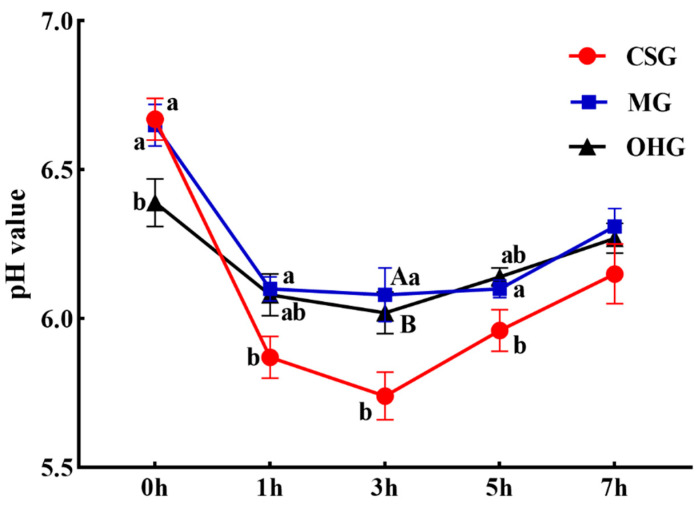
The dynamic changes of the sheep rumen liquid pH value over time. Different lowercase letters in the same industry indicate significant differences (*p* < 0.05), and different uppercase letters indicate extremely significant differences (*p* < 0.01).

The pH of the rumen fluid of the three groups first decreased and then increased over time. Specifically, it was highest before the morning feeding (0 h), began to decrease, and continued to decrease until 3 h after the morning feeding. After the pH reached its lowest value, it began to rise. The pH of the CSG was the highest before the morning feeding and was significantly higher than that of the OHG (*p* < 0.05). One hour and 5 h after feeding, the pH in the CSG was significantly lower than that in the OHG (*p* < 0.05), and, at 3 h, it was extremely significantly lower than that in the MG (*p* < 0.01) (Figure 1).

### 3.5. Microbial Alpha Diversity Analysis

A total of 1,593,972 pairs of reads were obtained by sequencing 74 samples, and a total of 158,685 clean tags were generated after the paired-end reads were spliced and filtered. Each sample generated at least 294 clean tags, with an average of 2144 clean tags (Table S1). When grouped at the 97% similarity level, 30,848 OTUs were identified in the 74 samples of bacteria. Intragroup analysis revealed that in the CSG, as the rumen digestion time increased, the number of OTUs increased, with the fewest OTUs at 0 h (473) and the most at 7 h (502). With an increase in rumen digestion time in the MG, the OTUs showed a trend of first decreasing and then increasing, while those in the OHG first increased and then decreased. Analysis among different groups showed that the CSG had the most OTUs (478), followed by the MG (405) and the OHG (384).

Alpha diversity is employed to analyze the species diversity of independent samples, including the Chao, Ace, Shannon, and Simpson indices, to measure species richness. The comparison within groups showed that microbial diversity and abundance in the CSG increased first and then decreased, while those in the MG and OHG decreased. The Shannon and Simpson indices of bacteria were 4.95 and 0.0029, respectively, in the CSG (0 h). The highest microbial diversity was found at 1 h; the Shannon and Simpson indices were 5.16 and 0.016, respectively. At 7 h, microbial diversity decreased, at which point the Shannon and Simpson indices were 5.03 and 0.022, respectively (Table 6). Comparisons among groups revealed that the OHG had the highest microbial diversity and abundance, with Shannon and Simpson indices of 5.31 and 0.014, respectively, and the CSG had the lowest microbial diversity and abundance, with Shannon and Simpson indices of 5.00 and 0.024, respectively (Table 7).

### 3.6. Microbial Abundance and Composition

**Figure 2:**
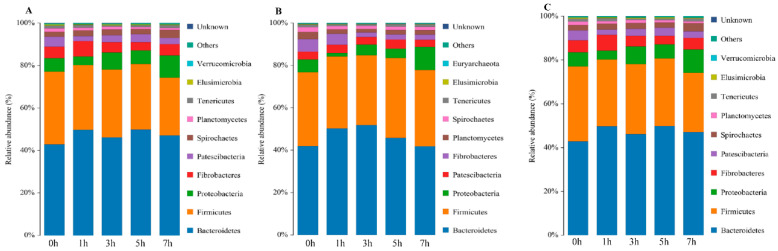
Community composition of bacteria at the tax level of phylum in different times. (**A**) CSG. (**B**) MG. (**C**) OHG.

**Figure 3:**
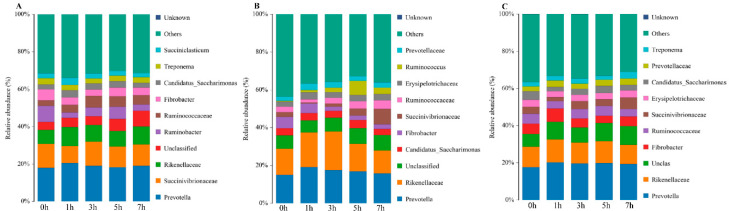
Community composition of bacteria at the tax level of genus in different times. (**A**) CSG. (**B**) MG. (**C**) OHG.

According to the phylum assignment results, Bacteroidetes was the main group of bacteria in the rumen fluid of sheep in the CSG; its abundance in this group was lowest before the morning feeding (0 h 35.5%) and highest at 7 h (43.5%). The next most common phylum in the CSG was Firmicutes, the abundance of which was lowest at 0 h (26.2%), increased at 1–3 h (approximately 30.4%), and decreased at 5–7 h (approximately 28.7%). Bacteroidetes was also the most important microbial phylum in the MG and OHG, where its abundance was highest at 3 h (51.9%) and 5 h (49.9%), respectively. The next most important phylum in the MG and OHG was Firmicutes, which was highest in abundance at 5 h (37.7%) and 0 h (34.2%), respectively (Figure 2). Below the phylum level, the most predominant taxonomic groups in the CSG were Prevotella, Succinivibrionaceae, and Rikenellaceae, which were most abundant at 1 h (20.6%), 3 h (12.8%), and 1 h (10.2%), respectively. The dominant subphylum groups in the MG were Prevotella and Rikenellaceae, which were most abundant at 1 h (19.1%) and 3 h (20.4%), respectively. The dominant flora in the OHG were also Prevotella and Rikenellaceae, both of which were most abundant at 1 h (20.2% and 12.4%, respectively) (Figure 3).

**Figure 4:**
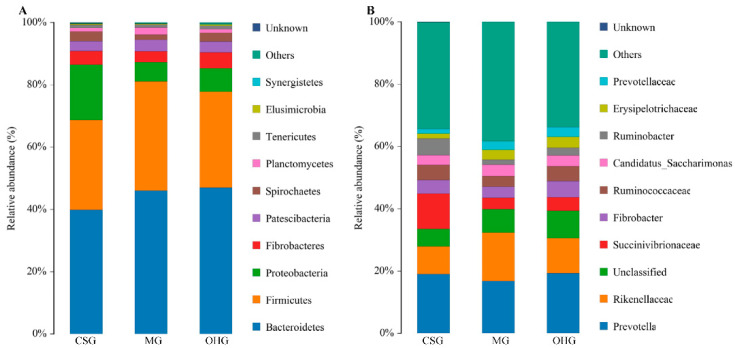
Community composition of bacteria in different groups. The tax level of phylum (**A**), the tax level of genus (**B**).

Comparisons among groups revealed that the dominant phyla of bacteria in the three groups were Bacteroidetes, Firmicutes, and Proteobacteria, but Bacteroidetes was the most abundant in the OHG (47.0%), Firmicutes was the most abundant in the MG (35.0%), and Proteobacteria was the most abundant in the CSG (17.8%). According to the subphylum assignment results, the dominant group in the CSG was Prevotella (19.0%), followed by Succinivibrionaceae (11.4%). Notably, when sheep were fed oat hay, the microbial community changed. The dominant group in the MG and OHG became Prevotella, followed by Rikenellaceae (Figure 4).

### 3.7. Microbial Communities

**Figure 5:**
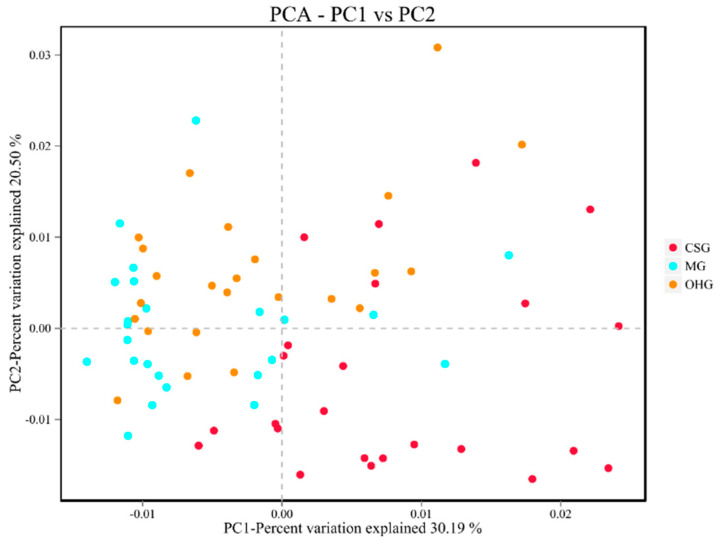
The effect of oat hay on the beta diversity of rumen microbes in sheep.

The differences between individuals or groups are reflected by the distances between sample points in the graph. The closer the samples in the graph, the greater the similarity is. As shown in Figure 5, the rumen microbial diversities of the MG and the OHG were similar, while the rumen microbial diversity of the CSG was quite different from those of the other two groups.

**Figure 6:**
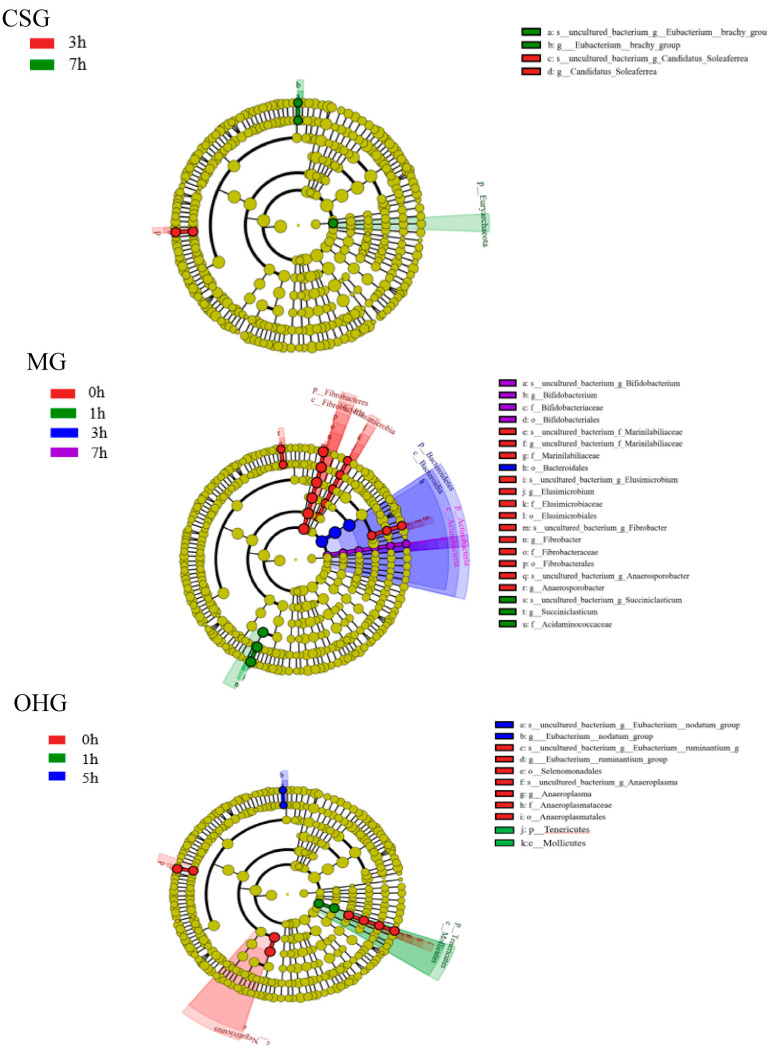
Linear discriminant analysis of effect size (LEfSe) was used to identify significant differences between bacterial groups in the same group at different times.

To determine the functional community/communities in samples, the LEfSe technique was conducted to identify the groups that displayed significant differences across the different bedding stages; the identified indicator groups are shown in a cladogram. For the CSG, there were two distinct microbial groups between 3 h and 7 h, namely Eubacterium and Candidatus, and there were no differences at the other time points. For the CSG, there were 14 groups with significant differences between 0 h, 1 h, 3 h, and 7 h. At 0 h, Marinilabiliaceae, Elusimicrobium, Elusimicrobiaceae, Elusimicrobiales, Fibrobacter, Fibrobacteraceae, Fibrobacterales, and Anaerosporobacter were abundant. Succiniclasticum and Acidaminococcaceae were abundant at 1 h. Bifidobacteriales was abundant in 3 h. Bifidobacterium, Bifidobacteriaceae, and Bacteroidales were abundant at 7 h. In the OHG, there were 8 distinct microbial groups at 0 h, 1 h, and 5 h. Among them, Eubacterium was abundant at 0 h; Eubacterium__ruminantium, Selenomonadales, Anaeroplasma, Anaeroplasmataceae, and Anaeroplasmatales were abundant at 1 h; and Mollicutes and Tenericutes were abundant at 5 h (Figure 6).

**Figure 7:**
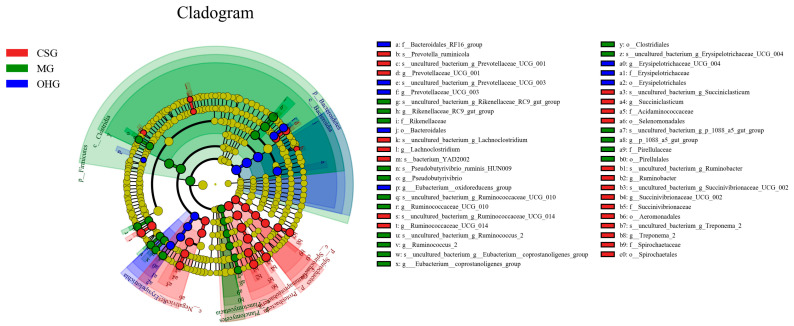
Bacterial taxa significantly differentiated between samples padded different groups identified by linear discriminant analysis effect size (LEfSe).

Comparisons among groups showed that there were 46 distinct microbial groups in the three feeding groups: Proteobacteria, Succinivibrionaceae, Gammaproteobacteria, and Aeromonadales were abundant in the CSG; Rikenellaceae, Firmicutes, Clostridia, and Clostridiales were abundant in the MG; and Bacteroidales, Bacteroidetes, and Erysipelotrichales were abundant in the OHG (Figure 7).

### 3.8. Association of Microbial Diversity and Abundance with Environmental Variables

**Figure 8:**
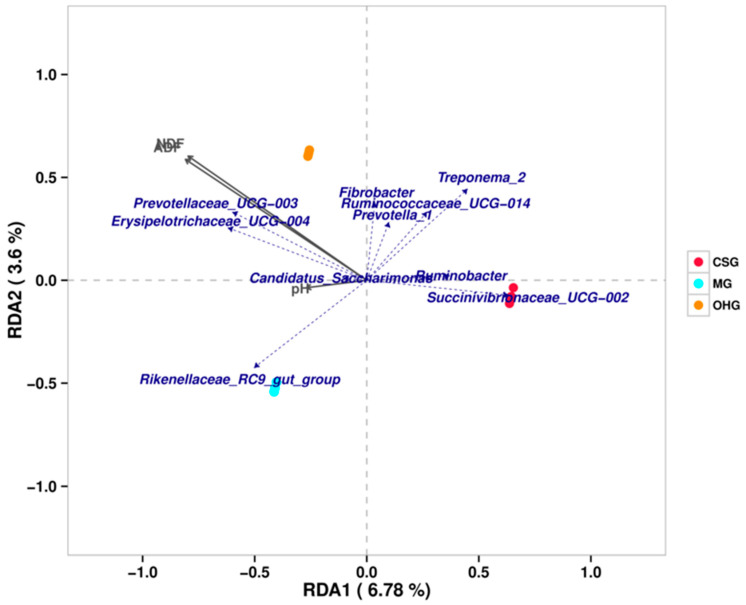
Bi-plot from the redundancy analysis (RDA) that shows the relationships between the bacterial community composition at the genus level and the environmental variables for all samples.

Three environmental factors (i.e., ADF, NDF, and pH) were found to be correlated with the microbial communities at the subphylum level by redundancy analysis (RDA). Axis 1 explained 6.87% of the variance, and axis 2 explained 3.6% of the variance. Prevotellaceae and Erysipelotrichaceae were positively correlated with pH, ADF, and NDF, and their scales were affected by these three environmental factors. Rikenellaceae exhibited a positive correlation with pH and was mainly affected by this environmental factor. The abundances of Fibrobacter, Ruminococcaceae, and Prevotella were mainly affected by the ADF and NDF in feed (Figure 8).

## 4. Discussion

### 4.1. Effect of Oat Hay Content in the Diet on Apparent Digestibility in Sheep

DM intake is an important indicator for measuring the production and health of ruminants, and the digestibility of DM and OM can reflect the digestive characteristics of an animal’s diet. For a diet with a concentrate content less than 10%, the DM intake of roughage will increase with an increase in the grain content in the concentrate, and the proportion of grain in the concentrate will increase from 10% to 70%, leading to a decrease in the DM intake of roughage [26]. Studies have also shown that the proportion of concentrate in diets is positively correlated with the apparent digestibility of DM [27]. In this study, although the differences in DM intake, digestion and apparent digestibility between the three groups were not significant, an increase in oat hay content in the diet tended to increase these metrics. Therefore, hay was beneficial for increasing DM intake, digestibility and apparent digestibility in sheep. In our study, the apparent digestibility of OM did not differ significantly among the three groups, which might have been due to the increased feed intake of the test sheep, while the retention time of various nutrients in the rumen was shortened, preventing those nutrients from being fully digested and absorbed. These results were consistent with those from other reports that different feeding levels have no significant impacts on the apparent digestibility of DM and OM [28].

Fibrous material is mainly degraded in the rumen of ruminants, and its fermentation products not only provide energy for ruminants and rumen microbes but also have important significance for ruminant saliva secretion, rumination, rumen buffering, and other processes. The digestibility of NDF and ADF can be used to measure the digestibility of diets in ruminants. It has been shown that the digestibility of OM will increase with an increase in the proportion of condensed materials, but the digestibility of NDF and ADF decreases with an increase in the proportion of concentrates [29]. In this study, the feed intake, digestibility, and apparent digestibility of NDF and ADF all increased significantly with the content of oat hay in the diet, indicating that feeding oat hay to sheep can help increase their appetite and intake. Feed intake, and thus the digestibility of crude fiber, also increased. However, some scholars have argued that feed intake is inversely proportional to nutrient digestibility [30,31].

The digestion and utilization of protein by ruminants were closely related to protein degradation by rumen microorganisms and the excretion of fecal nitrogen and UN. In this study, the intake of nitrogen by sheep increased significantly with an increase in the content of oat hay in the diet, but the content of oat hay in the diet had no significant effect on the apparent digestibility of nitrogen. One possible reason was that the available energy provided to the microbes by the diet was not coupled with the degradation of nitrogen, resulting in a decrease in the rate of feed nitrogen degradation. In this study, the excretion of nitrogen in feces was lower than that in urine, but both showed a decreasing trend with an increase in the oat hay content. It has been reported that the daily excretion of fecal nitrogen and of UN in sheep are not the same. Nitrogen retention could directly reflect the degree of utilization of dietary protein by the animal body, but nitrogen digestibility cannot reflect the degrees of digestion and absorption because ammonia in the rumen that cannot be used by microorganisms is absorbed by the rumen wall and enters the liver to be converted into urea. It is then excreted in the urine and largely lost. Therefore, the retention rate of nitrogen is more important than the digestibility of nitrogen [32]. In this study, both the deposited nitrogen and its deposition rate increased with an increasing oat content, indicating that replacing a certain proportion of whole corn silage with oat hay was beneficial to the utilization of dietary nitrogen.

### 4.2. Effect of Oat Hay Content in the Diet on Rumen pH

Rumen pH directly reflects the rumen fermentation level and internal environmental conditions of ruminants. The rumen fermentation level can be assessed by measuring rumen pH [33]. The cellulolytic bacteria in the rumen are active when the pH of the rumen fluid is above 6.30. A higher pH value is suitable for the reproduction of cellulolytic bacteria and the synthesis of protein by rumen microorganisms [34]. When the pH of rumen fluid drops from 6.8 to 5.8, the degradation time of NDF is prolonged, and the degradation rate decreases [35]. In this study, the pH of the rumen fluid was maintained between 5.8 and 6.7, and it decreased first and then increased with time in all three feeding groups. The pH was the highest at 0 h and decreased to its lowest value at 3 h after feeding. The pH of the rumen fluid in the CSG was slightly lower than 6.0 within 1–3 h, which may have been due to the digestion and decomposition of carbohydrates in the diet by rumen microorganisms to produce a large amount of volatile fatty acids (VFAs) [36]. As the diet was digested, buffer substances, such as saliva, increased, causing the rumen pH to slowly rise. The pH value of the rumen did not differ significantly among the three groups, indicating that the concentration of the concentrate had a small effect on the rumen pH value at 30–50% [34]. However, the rumen pH of the MG and the OHG was higher than that of the CSG, which may have been due to the oat hay having a higher structural carbohydrate content than the whole-plant corn silage, its palatability being relatively low, and its feeding rate being slower than those of the other diets [37].

### 4.3. Effects of Oat Hay Content in the Diet on the Rumen Microflora of Sheep

In this study, the Illumina HiSeq sequencing platform was applied to study the effects of different additions of oat hay on the rumen microflora of sheep. The Ace and Chao indices represent the relative abundance of microbial communities [38]. In this study, as the content of oat hay in the roughage increased, the Ace and Chao indices increased, and the Ace and Chao indices of the OHG were significantly higher than those of the CSG. This showed that the type of roughage in the diet affects the abundance of rumen bacteria, and an increase in the content of oat hay in the diet will increase the abundance of rumen bacteria. The larger the Shannon index value is, the smaller the Simpson index value, indicating a higher species diversity in the sample [39]. In this study, the Shannon index of the OHG was significantly higher than that of the CSG, while the Simpson index was significantly lower, indicating that the higher the content of oat hay in the sheep diet was, the higher the diversity of the rumen bacteria.

This study showed that Bacteroidetes and Firmicutes were the most abundant microbial phyla in the rumen flora, and Proteobacteria was the third most abundant microbial phylum, with a much lower abundance than Bacteroidetes and Firmicutes [40,41]. The number of microbial taxa in the rumen was affected by diet structure [42]. The relative abundance of Bacteroidetes did not differ greatly among the three groups, with values of 28.7%, 35.0%, and 30.7%. The relative abundance of Firmicutes in the CSG was significantly lower (*p* < 0.05) than that in the other two groups, while the relative abundance of Proteobacteria was significantly higher (*p* < 0.05). This indicated that increasing the content of oat hay in the diet increases the relative abundance of Firmicutes and decreases the relative abundance of Proteobacteria. The results showed that Firmicutes and Proteobacteria in the rumen of ruminants were closely related to the type of roughage in the diet.

When studying the effect of feeding different roughages on sheep rumen bacteria, it was found that high-fiber diets did not affect the total number of bacteria in the rumen but increased the number of fiber-degrading bacteria. Under diets consisting of very coarse feed, acid Vibrio and Prevotrine were the dominant bacteria in the rumen, and the number of Fibrobacteres in the rumen tended to increase [43,44]. Prevotella has the ability to degrade hemicellulose or xylan [45]. The metabolites are mainly acetic acid, succinic acid, and formic acid. Therefore, an increase in the abundance of this genus might make oat hay feeding possible, and the content of acetic acid in the rumen is reduced after grain feeding. Studies have revealed that the genus Pellegrini is closely related to the degradation of pectin and can interact with cellulolytic bacteria to participate in the degradation of cellulose [46]. Cellobacterium was the only genus in the phylum Firmicutes detected in a previous study [47]. The abundance of cellobacteria in the OHG was higher than that in the other two groups, indicating that the rumen environment of lambs was conducive to the growth of cellobacteria under oat hay diet feeding.

## 5. Conclusions

Feeding oat hay as coarse fodder significantly increased the intake, digestibility, and apparent digestibility of ADF and NDF in sheep and improved the body’s absorption and utilization of nitrogen. At the same time, feeding oat hay diet makes the pH value of sheep rumen fluid within the normal range, the rumen microbial abundance is high, the content of Prevotella and Fibrobacter in the rumen increases, and it promotes the decomposition of carbohydrate and dietary cellulose in the rumen. The oat hay diet not only improved the apparent digestibility and maintained the stability of the rumen environment but also significantly increased the species richness and diversity of rumen microbes. It is recommended to add a certain amount of oat hay to the sheep’s diet to increase the feed conversion rate and apparent digestibility.

## Figures and Tables

**Table 1:** The experimental design for evaluating 3 kinds of roughage using 9 fistulated rams.

Treatments	Fistulated Ram								
	Square 1			Square 2			Square 3		
	1	2	3	4	5	6	7	8	9
Period 1	CS	M	OH	OH	M	CS	M	OH	CS
Period 2	M	OH	CS	CS	OH	M	OH	CS	M
Period 3	OH	CS	M	M	CS	OH	CS	M	OH

**Table 2:** Composition and nutrient levels of experimental diets (dry matter (DM) basis) %.

Items	Whole Corn Silage Group (CSG)	Mixed Group (MG)	Dried Oat Hay Group (OHG)
Ingredients			
Dried oat hay	0	32.75	65.50
Whole corn silage	65.50	32.75	0
Corn	25.74	28.67	30.42
Soybean meal	2.34	1.75	1.18
Rapeseed meal	2.34	1.17	0.58
Cottonseed meal	2.24	1.17	0.58
Limestone	0.58	0.58	0.58
NaCl	0.58	0.58	0.58
Premix ^1^	0.58	0.58	0.58
Total	100.00	100.00	100.00
Nutrient levels			
Digestive energy DE (MJ/kg)	18.39	18.29	18.01
Crude protein CP	18.13	17.84	17.73
Calcium Ca	0.41	0.46	0.39
Total phosphorus TP	0.27	0.28	0.26
Neutral detergent fiber NDF	58.12	63.79	69.45
Acid detergent fiber ADF	38.23	38.52	38.79

^1^ The Premix provided the following per kg of diets: VA 220000 IU, VD3 72000 IU, VE 2000 IU, D-biotin 40.0 Mixed group, nicotinic acid amide 2000 Mixed group, Mn (as manganese sulfate) 710 Mixed group, Zn (as zinc sulfate) 2005 Mixed group, Fe (as ferrous sulfate) 830.0 Mixed group, Cu (as copper sulfate) 680.0 Mixed group, and Go (as Cobalt sulfate) 12 Mixed group. Nutritional level was measured.

**Table 3:** Effects of dried oat hay content in diet on DM and organic matter (OM) apparent digestibility of sheep.

Items	CSG	MG	OHG
Dry matter			
Intake/(kg/d)	1.62 ± 0.02	1.64 ± 0.01	1.66 ± 0.02
Fecal output/(kg/d)	0.58 ± 0.06	0.51 ± 0.05	0.48 ± 0.02
Digestion/(kg/d)	1.04 ± 0.07	1.13 ± 0.06	1.18 ± 0.03
Apparent digestibility/%	63.99 ± 3.83	68.64 ± 3.16	71.00 ± 1.15
Organic matter			
Intake/(kg/d)	1.46 ± 0.02 ^b^	1.50 ± 0.01 ^ab^	1.52 ± 0.02 ^a^
Fecal output/(kg/d)	0.52 ± 0.05 ^a^	0.44 ± 0.04 ^ab^	0.38 ± 0.01 ^b^
Digestion/(kg/d)	0.94 ± 0.06	1.05 ± 0.05	1.13 ± 0.03
Apparent digestibility/%	64.12 ± 3.81	70.31 ± 2.99	74.56 ± 1.01

In the same row, with different small letter superscripts mean significant difference (*p* < 0.05), the same as below.

**Table 4:** Effects of dried oat hay content in diet on neutral detergent fiber (NDF) and acid detergent fiber (ADF) apparent digestibility of sheep.

Items	CSG	MG	OHG
NDF			
Intake/(kg/d)	0.53 ± 0.01 ^B^	0.55 ± 0.01 ^AB^	0.56 ± 0.01 ^A^
Fecal output/(kg/d)	0.32 ± 0.03 ^a^	0.27 ± 0.02 ^ab^	0.23 ± 0.01 ^b^
Digestion/(kg/d)	0.21 ± 0.04 ^b^	0.28 ± 0.03 ^ab^	0.33 ± 0.01 ^a^
Apparent digestibility/%	40.10 ± 6.37 ^b^	51.69 ± 4.87 ^ab^	58.36 ± 1.65 ^a^
ADF			
Intake/(kg/d)	0.29 ± 0.01 ^Bb^	0.30 ± 0.01 ^a^	0.31 ± 0.01 ^A^
Fecal output/(kg/d)	0.18 ± 0.02 ^a^	0.16 ± 0.01 ^ab^	0.14 ± 0.01 ^b^
Digestion/(kg/d)	0.11 ± 0.02 ^b^	0.14 ± 0.02 ^ab^	0.17 ± 0.01 ^a^
Apparent digestibility/%	36.65 ± 6.74 ^b^	47.61 ± 5.28 ^ab^	55.71 ± 1.75 ^a^

In the same row, with different small letter superscripts mean significant difference (*p* < 0.05), and with different capital letter superscripts mean extremely significant difference (*p* > 0.01), the same as below.

**Table 5:** Effects of dried oat hay content in diet on N apparent digestibility of sheep.

Items	CSG	MG	OHG
N Intake/(g/d)	35.79 ± 0.37 ^b^	36.45 ± 0.28 ^ab^	37.56 ± 0.39 ^a^
Fecal N/(g/d)	12.56 ± 1.23	11.57 ± 1.08	10.35 ± 0.33
Urinary N	16.02 ± 0.16	16.21 ± 0.12	15.86 ± 0.16
N Digestion/(g/d)	23.23 ± 1.57	24.88 ± 1.36	27.21 ± 0.66
Apparent digestibility/%	64.82 ± 3.74	68.21 ± 3.20	72.42 ± 1.09
Retained N/(g/d)	7.21 ± 0.52 ^b^	8.67 ± 0.38 ^ab^	11.35 ± 0.64 ^a^
Retained N/N Intake/%	20.15 ± 1.26 ^b^	23.78 ± 1.34 ^ab^	30.22 ± 1.56 ^a^

In the same row, with different small letter superscripts mean significant difference (*p* < 0.05), the same as below.

**Table 6:** Paired-end reads and alpha diversity of sheep rumen fluid bacterial community at different times.

Sample ID	Time (h)	PE Reads	OUTs	Ace	Chao	Shannon	Simpson
CSG	0 h	23,716 ± 203	473 ± 31	715.17 ± 58.90	617.70 ± 38.04	4.94 ± 0.15	0.029 ± 0.008
	1 h	24,005 ± 340	440 ± 17	678.22 ± 71.18	604.81 ± 40.52	5.16 ± 0.09	0.016 ± 0.003
	3 h	26,888 ± 390	480 ± 29	635.60 ± 48.86	563.61 ± 39.61	4.99 ± 0.06	0.021 ± 0.003
	5 h	26,331 ± 176	497 ± 16	683.50 ± 48.10	585.481 ± 59.14	4.91 ± 0.03	0.032 ± 0.002
	7 h	29,825 ± 593	502 ± 37	648.18 ± 23.83	594.20 ± 37.06	5.03 ± 0.15	0.022 ± 0.006
MG	0 h	17,083 ± 437	424 ± 12	800.25 ± 67.12	678.10 ± 41.31	5.33 ± 0.03	0.011 ± 0.001
	1 h	16,287 ± 995	373 ± 26	651.59 ± 46.56	547.76 ± 28.15	5.20 ± 0.06	0.011 ± 0.001
	3 h	1614 ± 788	390 ± 28	735.45 ± 77.52	604.00 ± 47.41	5.15 ± 0.08	0.013 ± 0.001
	5 h	17,750 ± 227	383 ± 14	733.02 ± 79.63	626.36 ± 19.31	5.07 ± 0.06	0.016 ± 0.001
	7 h	25,173 ± 338	450 ± 20	706.09 ± 44.27	665.51 ± 20.70	5.15 ± 0.07	0.016 ± 0.002
OHG	0 h	21,085 ± 911	398 ± 30	717.71 ± 39.35	691.84 ± 30.90	5.40 ± 0.09	0.011 ± 0.002
	1 h	22,931 ± 647	411 ± 36	836.26 ± 68.34	672.92 ± 70.67	5.30 ± 0.10	0.013 ± 0.002
	3 h	20,791 ± 473	357 ± 31	780.04 ± 53.45	721.84 ± 28.92	5.29 ± 0.06	0.014 ± 0.001
	5 h	21,177 ± 94	386 ± 16	755.74 ± 26.29	744.72 ± 29.51	5.34 ± 0.06	0.014 ± 0.001
	7 h	17,408 ± 603	370 ± 34	782.50 ± 45.68	738.80 ± 45.82	5.25 ± 0.05	0.016 ± 0.001

**Table 7:** PE reads and alpha diversity of sheep rumen fluid bacterial community at different groups.

Sample ID	PE Reads	OUTs	Ace	Chao	Shannon	Simpson
CSG	26,153 ± 1109	478 ± 11	672.13 ± 22.01	593.21 ± 18.21	5.00 ± 0.06	0.024 ± 0.003
MG	18,487 ± 369	405 ± 14	724.96 ± 27.80	624.26 ± 17.15	5.18 ± 0.03	0.014 ± 0.001
OHG	20,678 ± 899	384 ± 10	771.87 ± 20.55	715.74 ± 17.67	5.31 ± 0.03	0.014 ± 0.001

## Supplementary Materials

The following are available online at https://www.mdpi.com/2076-2615/10/12/2341/s1, Table S1: The original measurement results of all samples. The microbial sequences in our manuscript have been deposited in the SRA of the NCBI, accession number PRJNA669237. Biosample accession SAMN16449931-16450004.
